# Optimizing cell arrays for accurate functional genomics

**DOI:** 10.1186/1756-0500-5-358

**Published:** 2012-07-17

**Authors:** Sven Fengler, Philippe I H Bastiaens, Hernán E Grecco, Pedro Roda-Navarro

**Affiliations:** 1 Department of Systemic Cell Biology, Max Planck Institute for Molecular Physiology, Dortmund, Germany; 2 Immunology Research Institute Hospital 12 de Octubre (i+12), Faculty of Medicine, Universidad Complutense de Madrid, Madrid, Spain

**Keywords:** Automated microscopy, Cell arrays, Methods for systems biology, Reverse transfection, Single cell analysis

## Abstract

**Background:**

Cellular responses emerge from a complex network of dynamic biochemical reactions. In order to investigate them is necessary to develop methods that allow perturbing a high number of gene products in a flexible and fast way. Cell arrays (CA) enable such experiments on microscope slides via reverse transfection of cellular colonies growing on spotted genetic material. In contrast to multi-well plates, CA are susceptible to contamination among neighboring spots hindering accurate quantification in cell-based screening projects. Here we have developed a quality control protocol for quantifying and minimizing contamination in CA.

**Results:**

We imaged checkered CA that express two distinct fluorescent proteins and segmented images into single cells to quantify the transfection efficiency and interspot contamination. Compared with standard procedures, we measured a 3-fold reduction of contaminants when arrays containing HeLa cells were washed shortly after cell seeding. We proved that nucleic acid uptake during cell seeding rather than migration among neighboring spots was the major source of contamination. Arrays of MCF7 cells developed without the washing step showed 7-fold lower percentage of contaminant cells, demonstrating that contamination is dependent on specific cell properties.

**Conclusions:**

Previously published methodological works have focused on achieving high transfection rate in densely packed CA. Here, we focused in an equally important parameter: The interspot contamination. The presented quality control is essential for estimating the rate of contamination, a major source of false positives and negatives in current microscopy based functional genomics screenings. We have demonstrated that a washing step after seeding enhances CA quality for HeLA but is not necessary for MCF7. The described method provides a way to find optimal seeding protocols for cell lines intended to be used for the first time in CA.

## Background

Cellular responses are achieved by complex intracellular biochemical networks that integrate signals transduced from the extracellular environment. Acquiring functional understanding of such processes requires identifying main components of these networks and determining their dynamic functional connections. Due to the large number of gene products involved in any given cellular function, systemic approaches that combine assay miniaturization, sample quality control and acquisition automation are demanded.

In the last decade, automated quantitative microscopy has entered the *-omics era*. Among other detection methods, microscopy is unique to quantify processes in the cell, as it can provide high temporal and spatial resolution [1]. High throughput microscopy-based genomic screenings requires an addressable array of genomic material (plasmid DNA or siRNA) on imaging compatible cell culture chamber. Transfection in multi-well plates provides strict separation between samples but hinders the application of homogeneous treatment to all cells. By contrast, reverse transfection of immobilized genetic material arrayed in a single culture chamber allows spatially restricted perturbation of cell colonies without the use of wells. As all cells are grown in the same cell culture chamber, applied treatments are ineluctable homogeneous except for the unique transfection in each spot. In addition, all the experimental error generated due to well-to-well variation is avoided. By using these so called cell arrays (CA) the function of many proteins can be analyzed in a short time in a slide with up to 10 spots / mm^2^ (spot size 120-150 *μ*m in diameter). Thus, CA have emerged as new devices for automated high throughput quantitative microscopy providing subcellular and spatial resolution [2-4].

CA have been used to study genes involved in diverse cellular processes including apoptosis [5,6], subcellular localization [7-9], post-translational modifications [2,10], secretory pathways [11,12], identification of drug targets [2], signal transduction and transcriptional regulation [13-17]. Beyond cell-based phenotypic readouts cell array approaches have been successfully combined with fluorescence imaging techniques able to generate quantitative data about the molecular state of signaling network components on the single cell level [10].

Improved protocols for achieving optimal reverse transfection efficiencies and an optimal spot shape have been recently contributed [5,18-21]. However, a critical methodological aspect of CA is the fact that cell colonies separated by few microns are locally transfected while they are growing in the same cell culture chamber. Thus one of the major challenges is to find suitable protocols that accomplish a low rate of contamination among the genetic content present in different spots. In spite of the high number of cell-based screenings developed to investigate the function of gene products, quantitative cell-based data about the CA accuracy (intended as the capability of reverse transfection to generate desired patterns of expressed fluorescent proteins) is still not available.

**Figure 1:**
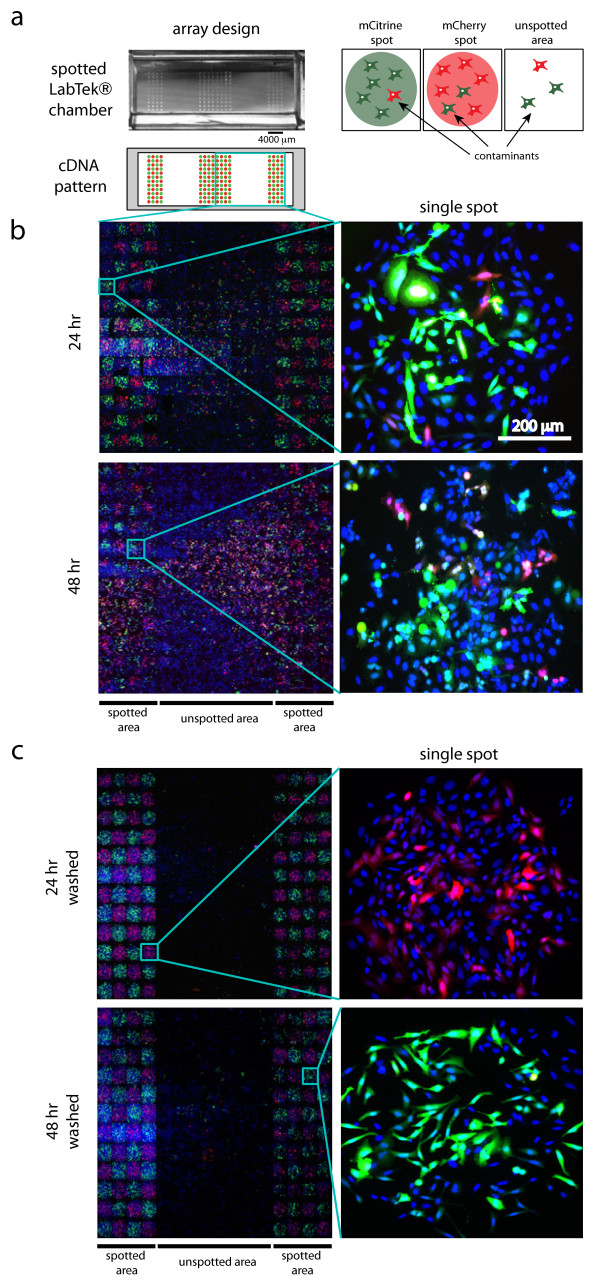
**Reduction of interspot contamination in HeLa cell arrays.** (**a**) Picture of a spotted LabTek chamber. Schematic of locations of plasmids encoding for mCitrine and mCherry (green and red spots), and unspotted (empty) areas are shown in the lower panel. Contaminants expressing the opposite fluorescent protein in mCitrine/mCherry spots or in unspotted areas are shown in the right schematic. (**b**) and (**c**) Montage of fields (spots or empty areas) of half arrays containing mCitrine/mCherry-expressing HeLa cells. 20 min after cell seeding a wash step was included in (**c**) but not in (**b**). Reverse transfection time is indicated. Right panels show magnifications of representative spots.

In this work, we quantified the percentage of contaminating cells in CA (Figure 1a). Surprisingly, presence of contaminating HeLa cells was a common feature of CA developed under standard protocols. We proved that the percentage of contaminating HeLa cells was 3 fold reduced when arrays were washed after cell seeding. Notably, interspot contamination was barely detected when using MCF7 cells, which demonstrate the suitability of these protocols for certain cell lines. By tracking cellular dynamics we demonstrated that cells mainly resided inside spots up to 48 hours analyzed, suggesting that directed cell migration among neighboring spots was not the major source of contamination. Thus, we propose that contaminating cells acquire genetic content during initial hours after the cell seeding before reaching the eventual spot address.

## Results and discussion

### Early washing after cell seeding increases cell array accuracy

We evaluated the accuracy of HeLa cell arrays as it is one of the most frequently used cell line in microscopy-based gene discovery programs [3,4,7,12,18,19,21-25]. Additionally we compared with MCF7 cells, a breast cancer model that has also been used in CA-based approaches [7,10].

For both cell types, we generated checkered arrays by printing mCitrine and mCherry expression plasmids in an alternate spot pattern. The expression of these chromophores after reverse transfection allowed us to monitor the transfection efficiency and the fraction of contaminating cells in each spot. In addition, part of the array was left unspotted to investigate the presence of transfected cells in those areas (Figure 1a).

We initially seeded cells under standard protocols [19]. After cell seeding, we incubated for 24 and 48 hours, commonly used for protein over expression after plasmid transfection and protein down-modulation after siRNA transfection, respectively. After 24 hours of incubation, mCitrine- and mCherry-expressing HeLa cells were distributed over the complete culture chamber, including empty areas where no spots were printed. After 48 hours, spots were poorly defined leading to undefined array grids (Figure 1b and Additional file 1: Figure S1). This data showed that accuracy of HeLa cell arrays was very low.

It was previously shown that the presence of fibronectin in the printing solution increases cell adherence [11]. We found that HeLa cells adhered faster on fibronectin containing spots, already 10-20 minutes after seeding, than on the glass surface, where it takes at least 40 minutes. This property prompted us to apply a washing step 20 minutes after cell seeding. In this way, mostly all non-adhered cells on the glass surface were washed away, while cells on spots stayed attached (Additional file 2: Figure S2).

To assess the effect of the washing step on cell array accuracy, we included a washing step after cell seeding. This modification of the protocol resulted in an improved expressed pattern. Cells on arrays were mainly located on spots while empty unspotted areas were mostly free of cells after both, 24 and 48 hours of reverse transfection (Figure 1c and Additional file 1: Figure S1). The distribution of cells after 48 hours in washed arrays indicates that cells do not migrate outside the spot during the reverse transfection time after cell attachment. Thus, data suggested that non-specific transfection of HeLa cells during the seeding process caused spot contamination.

**Figure 2:**
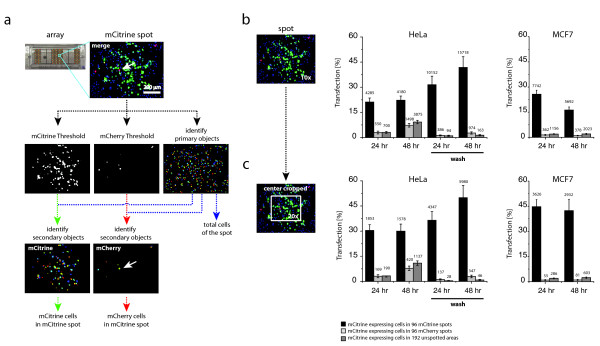
**Quantitative analysis of array accuracy.** (**a**) Schematic representation of automated image analysis. Schematic of the module built pipeline created with Cell Profiler software to calculate the numbers of mCitrine and mCherry expressing cells in mCitrine spots. Thresholds for mCitrine and mCherry channels were applied as described in methods. The total number of cells was calculated by identifying primary objects by using a nuclei staining. The threshold images of mCitrine or mCherry were used with the primary objects to identify mCitrine and mCherry expressing cells (secondary objects). The white arrow shows one of the interspot contaminating cells. (**b**) and (**c**) The percentage of mCitrine expressing cells was calculated for each mCitrine cDNA-containing spot (transfection efficiency, back bars) or for mCherry cDNA-containing spots (interspot contamination, light-gray bars). Dark-grey bars represent the % of mCitrine-expressing cells in empty areas. Results obtained with the protocol that includes the washing step are shown. An example of cropped areas used for 20x objective simulation is shown (left panels). The number of mCitrine cells and the standard deviation are indicated.

In order to quantify spot contamination, we segmented acquired images into single cells and measured the transfection efficiency and the percentage of contaminating cells in spots and empty areas (see methods, section 2.4) (Figure 2a and b). A 3% of contaminating HeLa cells after 24 hours of incubation was found. Similar values of contaminating cells were observed in images acquired from empty areas. After incubation times of 48 hours the percentage of contaminating cells in spots and empty areas reaches 7% and 9%, respectively. In contrast, a clear-cut reduction of the percentage of contaminating cells was found after washing steps. Less than 3% of contaminating cells were found even in 48 hours of incubation times (Figure 2b). Thus, washing steps increased notably the accuracy of cell arrays, which showed specific fluorescent protein patterns dictated by spot organization.

MCF7 cell arrays prepared using the standard protocol showed a percentage of contaminating cells in spots and empty areas under 2% for both incubation times (Figure 2b). In comparison with HeLa cells, this cell line was found to produce accurate cell arrays without the need of a washing step. This demonstrated that characterization and optimization of protocols for generating accurate CA with low contamination rates must be done for each cell line.

To further investigate how the contaminating cells are distributed, we cropped the center of acquired microscopy fields, where mainly the spot is located, and measured the percentage of transfected cells. The size of the cropped field simulated the magnification of a 20x objective (Figure 2c). These data showed a general increase of the transfection efficiency consistent with the fact that mainly non-transfected cells were located at the periphery of the spot. In contrast, the percentage of contamination showed no differences with respect to the un-cropped data, demonstrating that contaminating cells were not preferentially located at the spot periphery. As expected, transfection rate in empty areas did not show any change when cropped areas where analyzed. These data were consistent with the hypothesis that cell migration among spots is not the main reason of contamination which would render contaminant cells preferentially located at the spot periphery.

### Cell dynamics inside spots

**Figure 3:**
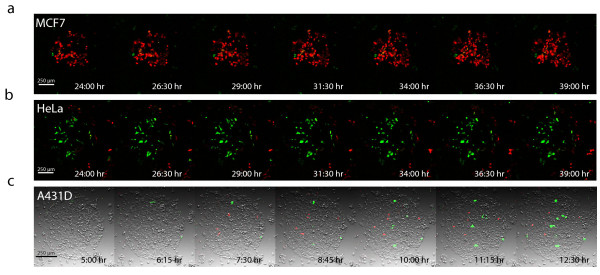
**Cell dynamics in spots.** MCF7 (**a**) or HeLa (**b**) cells seeded according to the standard protocol without washing step and incubated for 39 hours. After the first 24 hours of incubation cells were tracked in time lapse live cell imaging for 15 hours under growing conditions. The merged image (mCitrine in green and mCherry in red) of representative frames acquired every 2:30 hours are shown. (**c**) A431D cells were incubated for 5 hours after cell seeding and monitored for 7 hours. The merged image of the transmission, mCitrine (green) and mCherry (red) channels, taken every 75 min are shown.

In an attempt to further prove that cell migration among neighboring spots was not the main source of contaminating cells in cell arrays, live HeLa and MCF7 cells were seeded without a washing step, incubated for 24 hours and tracked in time-lapse microscopy experiments for 15 hours. We found that neither HeLa nor MCF7 cells moved away from spots during long incubation times (Figure 3a-b and Additional file 3 and Additional file 4). Moreover, experimentes where performed to track the appearance of fluorescence after chromophore expression and maturation. A431D cells tracked 5 hours after seeding captured the first detectable expression of mCherry in non-migrating cells located in mCitrine spots (Figure 3c and Additional file 5). This experiment indicated an early acquisition of the mCherry plasmid by these cells, before the final adhesion to the mCitrine spot where they were attached before fluorescence emission was detectable.

Presented data strongly support the hypothesis that cell migration is not the dominant source of contamination on cell arrays. We propose that contamination occurs at early times after cell seeding. Consistent with this hypothesis, this source of contamination can be notably reduced by applying a washing step early after seeding.

## Conclusion

CA have been used as platforms to analyze the function of thousands of genes with high efficiency in large gene discovery projects. Most of previous efforts to improve protocols to fabricate CA were focused on obtaining high reverse transfection efficiencies and better spot shape with different cell lines [5,18-21]. However, methods to quantify and correct the interspot contamination, susceptible to occur in an open device, have not been contributed so far.

In order to avoid interspot contamination it was suggested that the spot-to-spot distance could be increased [19]. The drawback of this strategy is that the total capacity of the array is reduced and a lower number of samples can be studied. Moreover, here we show that an increased spot-to-spot distance is not sufficient to reduce the contamination in the case of HeLa cells since fluorescent cells distribute to unspotted areas (Figure 1b). By contrast, protocols that include a washing step after seeding have been proved to be able to produce high density cell arrays with short spot-to-spot distances, good transfection efficiencies and spot shapes [26]. By using mCitrine and mCherry in an alternate spot pattern, we now show that these protocols not only produce CA with better spot shape but also with negligible contamination. This is particularly relevant in experiments done with siRNA where transfection times of 48 or 72 hours are applied.

Our results clearly indicate that migration on the substrate is not the major source of interspot contamination. First, we have shown that fibronectin added to the transfection mixture avoid the migration of transfected cells away from spots. Secondly, contaminating cells are not located at the spot periphery (Figure 2b and c). We then propose that contamination is originated early after cell seeding. It is possible that cells touch spots during seeding and get transfected before the eventual adherence at the final array address. The application of washing steps is a convenient way to reduce this phenomenon. This conclusion is further supported by tracking cells during early steps after cell seeding when the initial expression and maturation of chromophores can be observed (Figure 3c).

In contrast to the results generated with HeLa cells, MCF7 cells showed a 7-fold lower percentage of contaminating cells in cell arrays developed without washing steps after cell seeding. This demonstrates that contamination is not an universal problem and thus specific adhesion properties and transfection ability of different cell lines affect the quality of cell arrays developed under standard protocols.

A common aspect of cell-based assays is that cell-to-cell variability broadens the response to treatment, hindering the ability to detect significant changes. Control genes known not to affect cell phenotypes have been typically included to probe for this type of effects [19]. Contamination can increases the false positive rate, as foreign cells in a spot containing a gene unrelated to the observed phenotype might alter its observed distribution yielding an statistically significant difference with the control. However, in the common situation where most of screened gene products do not regulate the cell phenotype, contamination will increase not the false positive but the false negative rate, as most foreign cells will respond identically to the control.

In summary we have shown that the existence of contaminating cells in spots is a major caveat for certain cell lines and needs to be properly controlled. We described a quantitative method to measure the fraction of contaminating cells on arrays which should be used as a quality control to measure the suitability of cell lines intended to be use for the first time with this approach.

## Methods

### Cell culture

HeLa and MCF7 cells (ATCC), as well as A431D (kindly provided by Prof. Alpha Yap, Institute for Molecular Bioscience, University of Queensland, Australia) were cultured in DMEM (PAN Biotech GmbH) supplemented with 10% heat-inactivated fetal calf serum (FCS) (Invitrogen), 2 mM L-glutamine (PAN Biotech GmbH), non essential amino acids (PAN Biotech GmbH) and 100 U/ml penicillin and 100 *μ*g/ml streptomycin (Gibco) under 37°C and 5% CO_2_.

### Array production

Transfection mixtures containing mCherry (pmCherry-N1, Clontech) or mCitrine (pmCitrine-N1, A206K version of Citrine [27], kindly provided by Dr. Swanson, University of Michigan Medical School, MI) expression plasmids were done following the protocols previously described [19], and arranged in low volume 384 well source plates (Nalge Nunc International). Arrays containing 4 spotted areas of 4 rows and 12 columns with 1125 *μ*m distance between centers of every two neighboring spots, and 2 empty unspoted areas were developed by printing LabTek chambered cover glasses (Nalge Nunc International) with a robot spotter Qarray2 (Genetix) using solid pins (Array-It corporation, 500 *μ*m pin diameter) as previously described [28]. After printing, cell arrays dried for 12 hours in a box containing silica Gel Orange (Carl Roth GmbH) before cell seeding. Corner spots were labelled with a permanent marker for teaching their position to the microscope.

### Cell seeding

Before seeding, cells were washed with PBS, detached by 5 min Trypsin/EDTA (PAN Biotech GmbH), and resuspended in medium pre warmed at 37°C. 2.5*x*10^5^cells were dispensed on pre warmed arrays in a total volume of 3 ml complete medium. Cell arrays were incubated at 37°C and 5% CO_2_ for 24 or 48 hours to allow reverse transfection and protein expression.

As indicated, washes were done three times with warm medium 20 min after the same cell seeding procedure.

### Automated microscopy and data processing

After 24 or 48 hours incubation, cells were fixed in 4% paraformaldehyde/PBS (Sigma Aldricht) and washed with TBS (Tris.HCl 20mM, NaCl 150mM, pH 7.5, Takara Bio Europe). Cell nuclei were stained with 1 *μ*g/ml Hoechst (Sigma-Aldricht) in PBS after permeabilizing with 0.1% Triton X100/TBS (Serva). All arrays were automatically imaged using a fully motorized microscope (IX81, Olympus) with custom software [10]. Briefly, the microscope moved sequentially from spot to spot, following the predefined pattern printed by the spotter robot. A teaching step in which three spots are found in the microscope was performed before imaging to correlate the coordinate system of the robot and the microscope. At each spot on the array, Hoechst staining was used for auto-focusing, and images of Hoechst, mCitrine and mCherry channels were acquired and saved asynchronously. Filter sets for mCherry (U-MRFPHQ, Olympus), for mCitrine (U-MYFPHQ, Olympus) and for Hoechst (U-MNUA2, Olympus) were used.

Image processing and quantitative analysis was carried out using CellProfiler software [29]. The threshold for mCitrine was calculated by a two class Otsu-Global method. Threshold values of 24 images of mCitrine cDNA-containing spots were calculated according to this method. The average of resulting thresholds was subtracted by its standard deviation and applied to all acquired images. Images of the nuclear staining (Hoechst) were used to identify primary objects (total number of cells) by applying a two class Otsu per Object threshold. Identified objects (nuclei) were used to identify secondary objects by propagation in the mCitrine or mCherry channel. In this way, the total number of cells, the number of mCitrine and mCherry expressing cells were obtained for each spot. These values were first grouped according to the plasmid spotted (mCitrine, mCherry or untransfected). The mean value and standard deviation were then calculated for each group using bootstrap resampling [30] with 1000 repetitions using a builtin function in MatLab (Mathworks, USA). Fractional values were obtained by normalizing the number of transfected cells by the total number of cells (Figure 2b). In the second part, the center area (672 by 512 pixels) of each image (1344 by 1024 pixels) was cropped for analysis to simulate a higher magnification of 20x. Cropped images of all three channels were analyzed like described before.

### Live cell imaging

HeLa, MCF7 and A431D cells were seeded according the standard procedure, without applying any wash and incubated for 24 hours (HeLa and MCF7) or 5 hours (A431D). Cells were then imaged in DMEM without phenol red containing 25 mM HEPES (PAN Biotech GmbH) and 10% FCS at 37°C and 5% CO_2_. Time-lapse microscopy of single spots was performed with a confocal laser scanning microscope (FluoView 1000 Spectral, Olympus). A transmission image in conjunction with a fluorescence images of mCitrine and mCherry were obtained every 30 minutes for 15 hours (MCF7 and HeLa cells) and 7 hours (A431D). To image mCitrine and mCherry the sample was sequentially excited at 488 nm and 561 nm through a 488/561/633 dichroic. The emitted fluorescence was splitted using a dichroic mirror, spectrally filtered 515-560 (for mCitrine) or 580-610 (for mCherry) and detected with the internal PMTs set to analog mode.

## Abbreviation

CA: Cell Array.

## Competing interests

The authors declare no conflict of interest.

## Authors’ contributions

PRN and HEG share equal contribution as senior authors in research design, student supervision and manuscript writing. SF perform the experiments and analyzed the data. PIHB provided general project coordination and intellectual input. All authors revised the manuscript.

## Authors’ information

PRN is currently holding a research tenure track position at the Department of Microbiology I. Faculty of Medicine. Universidad Complutense de Madrid. Spain. email: proda@med.ucm.es

## Supplementary Material

Additional file 1 **Figure S1.** Arrays fabricated with HeLa cells. Complete cell arrays in LabTek chambers are shown. A washing step was applied as indicated (left). Cells were incubated for 24 hrs or 48 hrs to allow protein expression. Click here for file

Additional file 2 **Figure S2.** Effect of washing on cell seeding. HeLa cells seeded on arrays were washed 20 min or 40 min after seeding and immediately imaged under the microscope. Cell adherence on spots and the surrounding glass surface is compared. Magnification of spots acquired with 4x objective are shown (lower panel). Click here for file

Additional file 3 **HeLa cell dynamics on spots.** Time-lapse microscopy of HeLa cells transfected with the mCitrine plasmid printed in a spot. Image frames were obtained every 30 minutes after 24 hours of transfection time. Although cell migration is restricted to the area of the spot mCherry expressing cells (inter-spot contamination) can also be observed. Click here for file

Additional file 4 **MCF7 cell dynamics on spots.** Time-lapse microscopy of MCF7 cells transfected with the mCherry plasmid printed in a spot. Image frames were obtained every 30 minutes after 24 hours of transfection time. Cell migration is restricted to the area of the spot. Click here for file

Additional file 5 **Chromophore expression and maturation.** Time-lapse microscopy of A431D cells transfected with the mCitrine plasmid printed in a spot. Image frames were obtained every 15 minutes after 5 hours of transfection time. Expression of mCitrine is detected after 6 hours. mCherry-transfected cells resident in the spot are also detected. Click here for file
