# Supplementary figures and images for: Optimizing cell arrays for accurate functional genomics
Source: BMC Res Notes. 2012 Jul 17;5:358. doi: 10.1186/1756-0500-5-358 (PMC3541979; doi:10.1186/1756-0500-5-358)

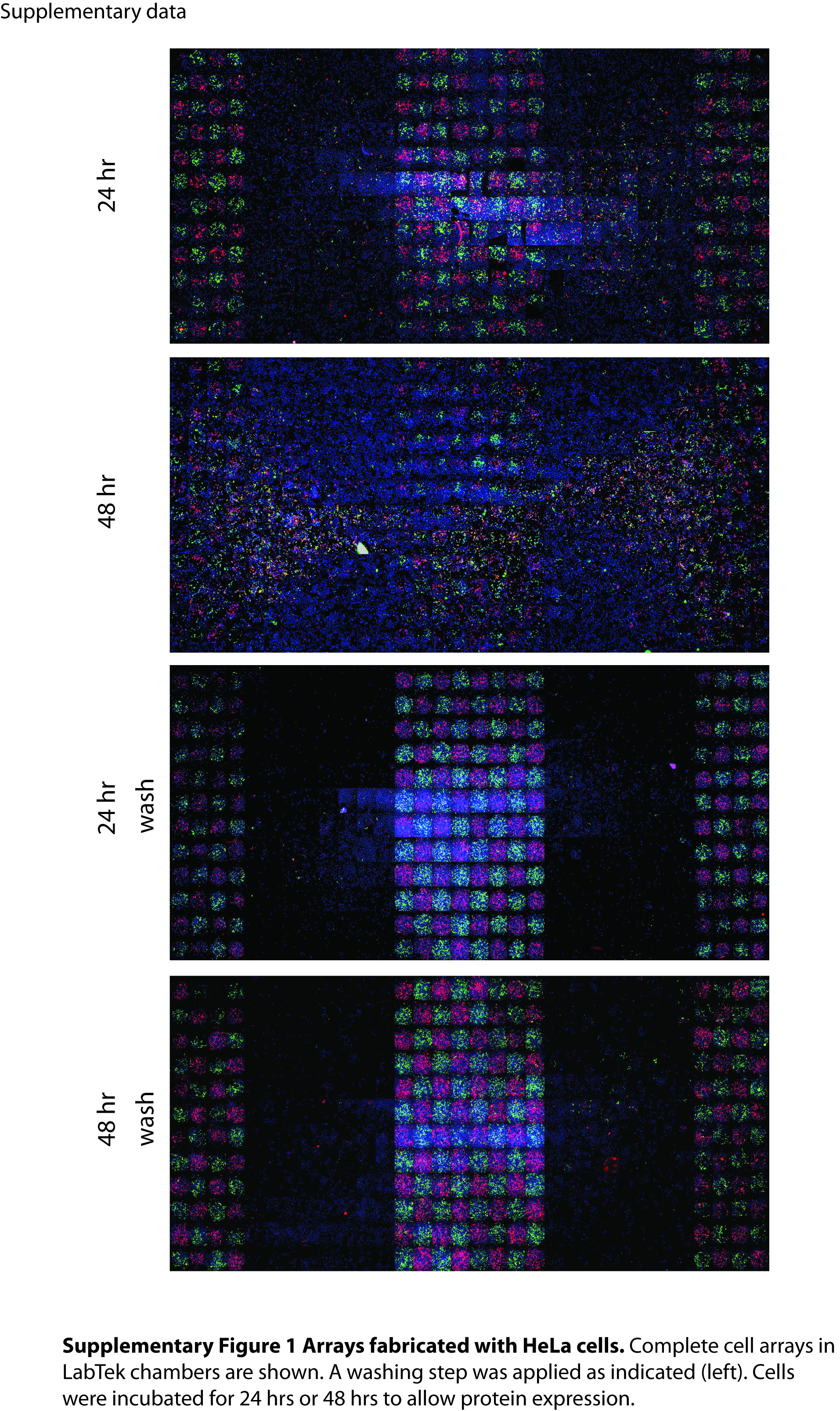

Supplement: Additional file 1 — Figure S1. Arrays fabricated with HeLa cells. Complete cell arrays in LabTek chambers are shown. A washing step was applied as indicated (left). Cells were incubated for 24 hrs or 48 hrs to allow protein expression. [file 1756-0500-5-358-S1.jpeg]

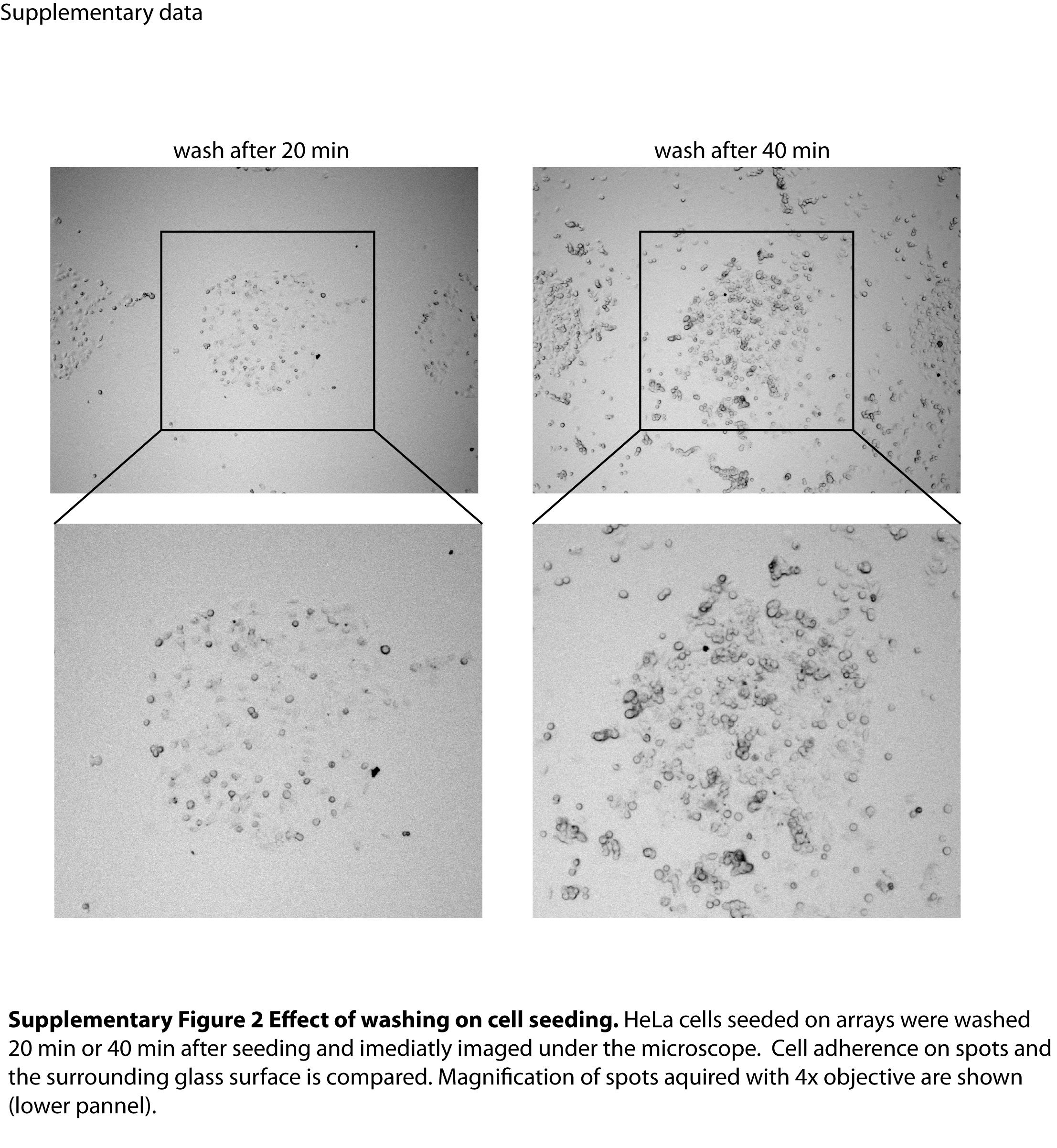

Supplement: Additional file 2 — Figure S2. Effect of washing on cell seeding. HeLa cells seeded on arrays were washed 20 min or 40 min after seeding and immediately imaged under the microscope. Cell adherence on spots and the surrounding glass surface is compared. Magnification of spots acquired with 4x objective are shown (lower panel). [file 1756-0500-5-358-S2.jpeg]
